# Is Silver Diamine Fluoride Effective in Arresting Enamel Caries? A Randomized Clinical Trial

**DOI:** 10.3390/ijerph19158992

**Published:** 2022-07-24

**Authors:** Araya Phonghanyudh, Duangporn Duangthip, Sirinan Mabangkhru, Varangkanar Jirarattanasopha

**Affiliations:** 1 School of Dentistry, King Mongkut’s Institute of Technology Ladkrabang, Bangkok 10520, Thailand; araya.ph@kmitl.ac.th; 2 Faculty of Dentistry, The University of Hong Kong, Hong Kong, China; dduang@hku.hk; 3 Faculty of Dentistry, Mahidol University, Bangkok 10400, Thailand; sirinan.mab@gmail.com

**Keywords:** silver diamine fluoride, fluoride varnish, enamel caries, early childhood caries, primary teeth

## Abstract

Background: There is limited information on the effectiveness of 38% silver diamine fluoride (SDF) in managing enamel caries. Objective: This study aimed to compare effectiveness of 38% SDF and 5% Sodium fluoride (NaF) varnish in arresting enamel caries in young children when applied semiannually over 18 months. Methods: A randomized controlled trial was conducted on children aged 1–3 years who had at least one active carious surface. They were allocated into two groups: Group 1 (38% SDF) and Group 2 (5% NaF varnish). Visual-tactile examination was used to assess extent of carious lesions. Enamel caries that did not progress to dentin were classified as having caries arrest. Intention-to-treat analysis was performed. Results: At baseline, 290 children with 1974 tooth surfaces with enamel caries were recruited. Caries arrest rates at the tooth surface level in Group 1 and Group 2 were 59.1% and 58.8%, respectively (*p* = 0.873), at 18 months. The multilevel logistic regression analysis revealed that tooth position, tooth surface, extent of enamel caries at baseline, caries experience, and brushing with fluoride toothpaste influenced caries arrest (*p* < 0.05). Conclusion: The semiannual application of 38% SDF and 5% NaF varnish had comparable effectiveness in arresting enamel caries in primary teeth.

## 1. Introduction

Early childhood caries (ECC) is one of the most important public health problems among young children. Children with ECC have a high caries risk and ECC can progress rapidly within 3–6 months of tooth eruption [1]. From 72 worldwide studies between 1998 and 2018, the mean prevalence rate of ECC at one year of age was approximately 17%, and this rate was projected to be higher than 50% at the age of five years [2]. The disease has a wide range of negative impacts on the quality of life of children, and the effects vary depending on the extent of the carious lesion [3,4]. Surgical management of ECC is very distressing not only for children but also for their families and dentists because of the limited cooperative ability of children. Moreover, this procedure requires sophisticated equipment and highly skilled dental teams. Alternative strategies that are simple and inexpensive are required to reduce the high prevalence of ECC in young children.

Some studies have shown that the progression of carious lesions at an early stage can be controlled by mineralization agents in conjunction with good oral hygiene and diet practices [5,6,7]. Early detection and timely management of the initial lesion can help reduce the burden of the disease. Among the mineralizing agents, fluoride is the most extensively used agent for the prevention and management of dental caries [5,8,9,10] and tooth erosion [11,12]. The mechanism of action of fluoride is the inhibition demineralization and enhancement of remineralization [13]. It is available in various forms, including toothpaste, mouth rinse, gel, and varnish, each with a different fluoride concentration and degree of clinical effectiveness [5,8,9,10,11].

Fluoride varnish is a professionally used topical fluoride preparation that is developed to prolong the contact time between fluoride and the tooth surface. Fluoride varnish usually contains 5% sodium fluoride (NaF) as the active component (22,600 ppm fluoride). Application of 5% NaF varnish is a simple, safe, painless, and relatively inexpensive intervention. Previous studies have investigated the clinical effectiveness of 5% NaF varnish in arresting the progression of enamel caries [5,10,14] and reported positive results regarding the use of 5% NaF for controlling the progression of non-cavitated enamel lesions in primary teeth [15,16].

Currently, silver diamine fluoride (SDF) is gaining popularity as an alternative agent for treating caries. It is a highly concentrated topical fluoride agent that conjugates with silver nitrate. It deteriorates the growth and vitality of cariogenic bacteria, impedes collagenase activity in dentine, inhibits demineralization, and promotes the remineralization process [17]. Several systematic reviews have confirmed the effectiveness of 38% SDF in arresting the progression of dentine caries lesions with a success rate of 65–91% [5,18]. It is widely used for caries control in community settings in many countries [19]. Although 38% SDF has been strongly recommended for treating dentine caries lesions, its effectiveness in arresting the progression of enamel caries lesions remains uncertain.

Therefore, this study was undertaken to evaluate the clinical effectiveness of semiannual applications of 38% SDF and 5% NaF varnish in arresting the progression of enamel caries in young children between 1–3 years of age who were at high risk of caries and residing in non-fluoridated areas over 18 months. The null hypothesis was that the caries arrest rates of carious enamel surfaces treated with 38% SDF were not statistically significant different from those of carious enamel surfaces treated with 5% NaF varnish. Parental satisfaction and adverse effects of these two interventions were also investigated.

## 2. Materials and Methods

This study was a part of the randomized clinical trial on caries arrest using silver diamine fluoride and fluoride varnish. The clinical trial was approved by the Institutional Review Board of the Faculty of Dentistry/Faculty of Pharmacy, Mahidol University (No. COA.No.MU-DT/PY-IRB 20181031.2205) and registered in the Thai Clinical Trials Registry (TCTR20180624001). The participants’ parents or guardians gave written informed consent prior to the start of the intervention.

The design of this study was a two-arm randomized controlled trial with interventions provided in a childcare setting. Participants who had at least one active caries were randomized into two intervention groups: Group 1 received 38% SDF (Topamine, DentaLife, Australia) and Group 2 received 5% NaF varnish (Duraphat, Colgate-Palmolive, Guildford, Surrey, UK). Both agents were applied semiannually to carious surfaces.

### 2.1. Sample Size Calculation

The estimated sample size was based on the result of the meta-analysis, concluding that the overall percentage of arrested dentin caries, which was the primary outcome in this clinical trial, was 65.9% [5], and an absolute 10% difference between groups was clinically significant. The power of the study was set at 80% (β = 0.2), with α = 0.05 as the statistical significance level. Thus, at least 343 dentin carious tooth surfaces were required in each arm, as calculated using Sample Power 2.0 (SPSS Inc., Chicago, IL, USA). The mean number of active dentin carious tooth surfaces was anticipated to be 7 [20]. The anticipated intraclass correlation coefficient (ICC) was 0.3 [21]. Based on the equation of sample size estimation in a multilevel study [22], 960 active carious tooth surfaces in 137 children/group would be required. With an estimated 10% dropout rate, approximately 150 children/group needed to be recruited at baseline.

### 2.2. Participants, Inclusion Criteria, and Setting

Participants of this study were recruited from 19 public child development centers located in a non-fluoridated area in Chiang Mai province, Thailand. Healthy children between 1–3 years of age who presented with at least one active dentine caries were invited to participate in this clinical trial. The study excluded children with no dentine caries, taking long-term medications or having a history of allergic reactions to fluoride, silver, or colophony agents.

Information on participants’ sociodemographic profiles and oral health-related practices was gathered using self-administered questionnaires, which were completed by their parents or guardians. A self-rated Likert scale was also used to indicate the satisfaction of parents or guardians with the dental appearance of their children. The data were collected at baseline and the 18-month follow-up visit.

Clinical examinations were performed by an examiner (S.M.) in the knee-to-knee position at the child development centers using World Health Organization CPI periodontal probes (405/WHO probe) with disposable dental mirrors attached to a light-emitting diode (LED) intraoral light source (MirrorLite, Kudos, Hong Kong, China). The examiner was trained for caries and plaque assessment by dental specialists in dental public health (D.D.) and pediatric dentistry (V.J.) until the weighted kappa values of inter-and intra-reliability were ≥0.8 each. Carious status was assessed according to the ICDAS criteria and WHO dmft (decayed, missing, and filled teeth) index. The visible plaque index (VPI), which is used to measure plaques on buccal and lingual surfaces of six index teeth (55, 51, 63, 71, 75, and 83), was used to determine the oral hygiene status of participants.

### 2.3. Randomization

The recruited participants were randomly allocated to one of the groups via stratified block randomization of four with two strata of the severity of caries experience (dmft = 1–4 and dmft > 4) through the generation of a random number table in the Excel program. Participant allocation lists were kept in sealed envelopes and arranged sequentially by an assistant who was not involved in the study. Each envelope was opened after completing an oral examination and immediately before the intervention.

### 2.4. Intervention

The intervention was performed by two dental nurses who were experienced in community dental programs. Before conducting the trial, they were trained by pediatric dentists (S.M. and V.J.) to apply SDF and NaF varnish on all carious surfaces in a community setting.

The clinical procedures of the research intervention were performed according to the following protocol: (1) all teeth were cleaned and isolated with gauze, and no attempt was made to remove carious tissues; (2) in Group 1, 38% SDF was directly applied and rubbed on each carious lesion using a disposable micro-applicator for 10 s, and in Group 2, 5% NaF varnish was applied to the lesions using the same type of micro-applicator; (3) after application, the participants were instructed to refrain from drinking, eating, and rinsing their mouth for at least 30 min; (4) the interventions in both groups were repeated every six months.

### 2.5. Outcome Assessment

Recall examinations were conducted at 6, 12, and 18 months after the first intervention to assess the effectiveness of both high concentration topical fluorides in arresting the progression of enamel carious lesions (ICDAS code 2 or 3). The enamel carious lesion was classified as arrested caries at follow-up examinations if the lesion did not progress to dentine (ICDAS code 4, 5, or 6). The examinations conducted at baseline and recall visits were performed by the same examiner, who was blinded to the group allocation and retrained prior to the recall visits. To assess intra-examiner reliability using weighted kappa statistics, duplicate examinations were performed on 10% of participants at baseline and follow-up examinations after two weeks of their initial examinations. Regarding intra-observer reliability, the kappa values for the duplicate assessment of the extent of caries lesions according to the ICDAS criteria were 0.86, 0.96, 0.90, and 0.86 at baseline and 6-, 12-, and 18-month follow-ups, respectively.

### 2.6. Data Analyses

Results were analyzed using SPSS version 20.0 for Windows (SPSS Inc., Chicago, IL, USA). The level of statistical significance was set at *p* < 0.05. Chi-square test was used to compare baseline sociodemographic profiles and oral health-related behaviors of participants, parental satisfaction, and enamel caries arresting rates between 38% SDF and 5% NaF varnish groups. Age, VPI scores, dmft, and dmfs scores were compared between both groups using an independent samples *t*-test or Mann–Whitney U test, depending on the normality of distribution.

Intention-to-treat analysis was performed. The carious lesion status of the lost participants was considered as caries progression. Carious surfaces that received restorative treatment and extraction between follow-up visits were recorded as caries progression. A multilevel multivariate logistic regression analysis was performed to analyze the effectiveness of 38% SDF in arresting the progression of enamel caries at the 18-month follow-up. Effects of potential variables, including baseline sociodemographic profiles, oral health-related behaviors, and clinical characteristics, were also evaluated.

Parental satisfaction with the dental appearance of children at baseline and 18-month follow-up and between the intervention groups were compared using the McNemar test and chi-square test, respectively.

## 3. Results

**Figure 1:**
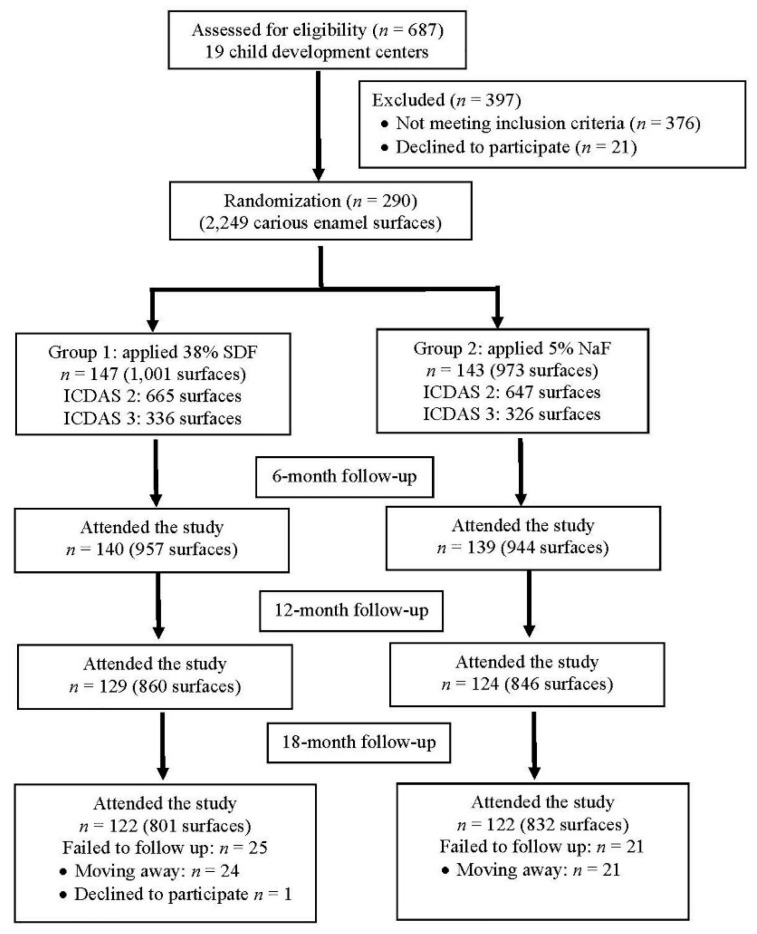
Flow of participants over the 18-month study.

In total, 302 participants participated in the clinical trial. There were 12 children (6 children in each intervention arm) who had no enamel caries and were excluded from this analysis. The balance between two groups among those who had enamel caries were observed. Group 1 had 147 participants with 1001 carious enamel surfaces who were treated with 38% SDF and Group 2 had 143 participants with 973 lesions who were treated with 5% NaF varnish (Figure 1). At the 18-month follow-up, 244 (84.2%) participants remained in the study. Dropout rates in Group 1 and Group 2 were 17.0% and 14.7%, respectively (*p* = 0.588). The main reasons for dropout were shifting to schools in other areas and family relocation to other communities.

The participants’ baseline socio-demographic background, oral health-related behaviors, and clinical characteristics are presented in Table 1. The mean (SD) age of the participants was 36.8 (6.5) months (range, 18–47 months). Socio-demographic profiles of both groups were comparable, except for the education level of mother (*p* = 0.014). Baseline oral health-related behaviors were not significantly different between the two groups (*p* > 0.05). The majority (>80%) of participants were bottle fed, and almost half of them continued bottle feeding while sleeping. Most participants brushed their teeth at least twice a day (90.0%) and used fluoride toothpaste (91.0%). There was no statistically significant difference in the baseline caries experience and VPI scores between Group 1 and Group 2. Overall mean dmft (SD) and dmfs (SD) scores were 5.4 (3.6) and 9.6 (9.1), respectively. Distributions of the non-cavitated enamel caries (ICDAS 2) and cavitated enamel caries (ICDAS 3) were similar between both groups.

Caries arrest rates at 6, 12, and 18 months are shown in Table 2. Overall proportions of arrested carious enamel surfaces in Group 1 were significantly higher than those in Group 2 at six months (*p* = 0.020) and 12 months (*p* = 0.024). However, after 18 months, there were no significant differences in caries arrest rates between Group 1 (59.1%) and Group 2 (58.8%) (*p* = 0.873). Caries arrest rates of non-cavitated enamel caries (ICDAS 2) were significantly higher than those of cavitated enamel caries (ICDAS 3) at all follow-up examinations, regardless of the intervention group.

Results of the multilevel logistic regression analysis of the 18-month follow-up data are presented in Table 3. The analysis demonstrated that 38% SDF and 5% NaF varnish had similar effectiveness in arresting the progression of enamel caries. Five factors influenced the progression of enamel caries: tooth position, tooth surface, the severity of enamel carious lesion at baseline, participant’s carious experience, and use of fluoride toothpaste. Non-cavitated enamel caries had a higher chance of remaining stable than cavitated enamel caries (OR = 2.89; 95% CI, 2.31–3.60). Enamel caries in children who brushed using fluoride toothpaste had a 2.39 times higher chance to be arrested compared with those in children who did not brush using fluoride toothpaste.

Regarding the side effects and esthetic concerns of using 38% SDF, no major adverse effects, including vomiting or nausea, were reported. At baseline, approximately 60% of parents were satisfied with the dental appearance of their children. At the 18-month follow-up, the satisfaction of the parents was similar to that reported at baseline in both groups (*p* > 0.05), and no significant difference was found between Group 1 and Group 2 (*p* > 0.05) (Table 4).

## 4. Discussion

It has been documented that 38% SDF is highly effective in arresting the progression of dentine caries in children [5,23,24]; however, evidence on its clinical effectiveness in arresting the progression of enamel caries is scarce. To the best of our knowledge, only one randomized controlled trial compared the effectiveness of 30% SDF and 5% NaF in arresting the progression of cavitated enamel caries (ICDAS 3) [24]. However, this study recruited only kindergarten children in a fluoridated area and used 30% SDF with intensive applications (applied three times at weekly intervals). Thus, there is limited information regarding the effectiveness of the semiannual application of 38% SDF in toddlers with high caries risk who live in a non-fluoridated area. Therefore, the results of the present study address this gap in the literature and are beneficial for clinical practitioners and public health workers in deciding whether to adopt 38% SDF or 5% NaF for controlling enamel caries in young children with high caries risk.

Based on data of 18 months of follow-up, caries arrest rates of 38% SDF and 5% NaF varnish applied semiannually were comparable; therefore, the null hypothesis of the study assuming no difference in enamel caries arrest rate between both interventions was accepted. These findings are in agreement with those of the study by Duangthip et al. [24]. Both 38% SDF and 5% NaF can be used to control the carious process by inhibiting demineralization and promoting remineralization. A possible explanation might be that silver and fluoride ions together may not have a better anticaries effect on enamel lesions compared to fluoride ions alone. A study by Liu et al. found that a high concentration of fluoride was effective in inhibiting demineralization of enamel, but treatment with silver ions alone (without fluoride) had little effect [25]. Possibly, fluoride ions act mainly on tooth substrate (demineralization and remineralization), whereas silver ions may affect mainly cariogenic bacteria in dentine caries, but not on precipitation of mineral ions in enamel.

The overall enamel caries arrest rate in our study was approximately 60%, which concurs with a systematic review [5]. The multilevel logistic regression analysis also confirmed that the extent of enamel caries at baseline influenced the caries arrest rates in the follow-up visits. The treatment effect on non-cavitated enamel caries (ICDAS 2) was higher than that on cavitated enamel caries (ICDAS 3). This highlights the importance of early intervention to control enamel caries effectively.

The results of multilevel logistic regression analysis demonstrated several significant variables, including tooth- and child-related factors influencing the progression of enamel caries, which were consistent with previous studies on factors arresting the progression of dentine caries [23,24,26]. Enamel caries that presented on the anterior teeth tended to be more controllable than those presented on the posterior teeth. Enamel caries on the buccal or lingual surface had a higher chance of getting arrested than caries on other tooth surfaces. Possibly, these areas are easily accessible for cleaning and are exposed to saliva and fluoride, thus increasing the remineralization process. Another significant finding was that enamel caries in children who brush their teeth using fluoride toothpaste was more likely to be arrested compared with those who brush using non-fluoride toothpaste. This result emphasizes the importance of encouraging parents and caregivers to brush their children’s teeth using fluoride toothpaste in conjunction with professionally applied topical fluorides.

Our study confirmed the safety of using 38% SDF in young children aged 1–3 years. No systemic adverse effects were reported during the 18-month study period. This was in line with a previous study conducted in children older than three years [27]. In our study, only one drop (25 µL) or less was used for each child, which contained 1.12 mg of fluoride and 6.34 mg of silver. The amounts of fluoride and silver used were much below the toxic dose [28,29]. Blackening or staining of carious lesions is a common side effect of SDF treatment, possibly affecting the satisfaction of parents and children. Interestingly, parental satisfaction with the dental appearance of children remained unchanged over 18 months. No significant differences were found between the two groups. This could be attributed to the effective communication and information provided to the parents during recruitment. Nevertheless, parental acceptance of SDF for caries control may vary in different cultures and contexts [30,31]. Dentists should inform parents regarding the advantages and disadvantages of using SDF for caries management.

As 5% NaF varnish and 38% SDF had similar treatment effects on the progression of enamel caries, both agents can be used for arresting enamel caries. For children who only have enamel caries, 5% NaF varnish would be a preferable alternative to avoid the black staining brought on by 38% SDF. Since 38% SDF is more effective in arresting dentine caries than 5% NaF varnish, it may be a better option for children with both enamel and dentine caries and no aesthetic concerns. Nevertheless, while selecting topical fluoride agents, additional aspects, including the cost of treatment, patient preference, and feasibility of use, should be considered.

Topical fluoride agents have been demonstrated preventing caries development and arresting initial carious lesion; however, their effectiveness have only been partially successful [5,14,16,24]. Because ECC is a multifactorial disease, preventing and controlling the progression of ECC cannot be solely based on a single intervention. It should be used in conjunction with basic preventive measures such as educating and encouraging parents or other caregivers to assist their children maintain good oral hygiene, limiting children’s sugar intake, and supplementing follow-up visits with caries risk assessments.

Our study had a few limitations. First, due to ethical concerns, there was no negative control group in this study. Second, the current study evaluated the enamel carious status by visual examination without radiographic examination. Some enamel carious lesions in the proximal areas that were not clinically detected were possibly not included in the study. Although a single examiner was blinded to the intervention groups, possible bias in the assessment of follow-up data might have occurred because of the staining of treated caries lesions caused by SDF. On the other hand, the current study has several strengths, including a low non-intervention-related dropout rate, sufficient sample size, and good intra-examination reliability. Nevertheless, this study was conducted among young children with high caries risk in an outreach setting. The results of the study may not be generalizable to other age groups or children with low caries risk. Further study on different groups of populations is required to elucidate or refute the effectiveness of SDF in arresting the progression of enamel caries.

## 5. Conclusions

Based on the 18-month results, there was no statistically significant difference in arresting enamel caries of primary teeth between the use of 5% NaF and 38% SDF applied semiannually in preschool children. The use of 38% SDF had no notable adverse effects and no impact on parental satisfaction with the dental appearance of children.

## Figures and Tables

**Table 1:** Socio-demographic background, oral health–related habits, and clinical characteristics of participants at baseline.

	Group 1: 38% SDF (*n* = 147)	Group 2: 5% NaF (*n* = 143)	*p*-Value
**Demographic background**	**Mean (SD)**	**Mean (SD)**	
Age(months) (min-max)	37 (7) (21–46)	37 (6) (18–47)	0.485
**Demographic background**	***n* (%)**	***n* (%)**	
Gender			0.577
Male	60 (40.8)	63 (44.1)	
Female	87 (59.2)	80 (55.9)	
Mother’s education level			0.014
Primary school or lower	74 (50.3)	94 (65.7)	
Secondary school	58 (39.5)	34 (23.8)	
College or university	15 (10.2)	15 (10.5)	
Monthly family income			0.841
<10,000 Baht	86 (58.5)	82 (57.3)	
≥10,000 Baht	61 (41.5)	61 (42.7)	
**Oral health-related habits**	***n* (%)**	***n* (%)**	
Daily milk feeding			0.939
None	27 (18.4)	27 (18.9)	
1–3 times	74 (50.3)	74 (51.7)	
>3 times	46 (31.3)	42 (29.4)	
Sleep with bottle			0.300
Yes	60 (40.8)	67(46.9)	
No	87 (59.2)	76 (53.1)	
Daily snack taking			0.154
<1 time	15 (10.2)	12 (8.4)	
1–2 times	104 (70.7)	90 (62.9)	
≥3 times	28 (19.0)	41 (28.7)	
Daily tooth brushing			0.697
<2 time	16(10.9)	13 (9.4)	
≥2 times	131 (89.1)	130 (90.9)	
Brushing with fluoride toothpaste			0.941
Yes	134(91.2)	130 (90.9)	
No	13 (8.8)	13 (9.1)	
**Clinical characteristics**	**Mean (SD)**	**Mean (SD)**	
dmft	5.0 (3.1)	5.8 (4.0)	0.247
dmfs	8.6 (7.1)	10.5 (10.7)	0.422
visible plaque index	0.7 (0.2)	0.7 (0.2)	0.526
**Caries characteristics**	***n* = 1001** ***n* (%)**	***n* = 973** ***n* (%)**	
Extent of caries			0.977
Non-cavitated enamel caries (ICDAS 2)	665 (66.4)	647 (66.5)	
Cavitated enamel caries (ICDAS 3)	336 (33.6)	326 (33.5)	
Tooth position			0.067
Upper anterior teeth	435 (43.5)	388 (39.9)	
Upper posterior teeth	218 (21.8)	242 (24.9)	
Lower anterior teeth	143 (14.3)	117 (12.0)	
Lower posterior teeth	205 (20.5)	226 (23.2)	
Tooth surface type			0.409
Buccal/lingual	750 (74.9)	709 (72.9)	
Proximal	66 (6.6)	61 (6.3)	
Occlusal	185 (18.5)	203 (20.8)	

**Table 2:** Caries arrest rate of enamel carious tooth surfaces at 6, 12, and 18-month follow-up examinations.

	Group 1: 38% SDF %(n/N)	Group 2: 5% NaF %(n/N)	*p*-Value
Overall			
6 months	86.5 (866/1001)	82.7 (805/973)	0.020
12 months	68.5 (686/1001)	63.7 (620/973)	0.024
18 months	59.1 (592/1001)	58.8 (572/973)	0.873
Non-cavitated enamel caries (ICDAS 2)			
6 months	92.6 (617/666)	90.6 (587/648)	0.178
12 months	77.9 (519/666)	73.3 (475/648)	0.051
18 months	68.2 (454/666)	69.6 (451/648)	0.576
Cavitated enamel caries (ICDAS 3)			
6 months	74.3 (249/335)	67.1 (218/325)	0.041
12 months	49.9 (167/335)	44.6 (145/325)	0.178
18 months	41.2 (138/335)	37.2 (121/325)	0.297

**Table 3:** Final logistic regression model of the caries arrest rate at the 18-month follow-up with the clustering effect adjusted.

Explanatory Variables	Adjusted Odds Ratio ^a^	95% CI	*p*-Value
**Group**			
38% SDF	1.13	0.79–1.60	0.501
5% NaF		1	
**Tooth position**			
Lower anterior	1.83	1.29–2.61	0.001
Lower posterior	0.77	0.58–1.01	0.060
Upper posterior	1.02	0.77–1.34	0.909
Upper anterior		1	
**Tooth surface type**			
Buccal/lingual	1.47	1.10–1.95	0.009
Proximal	0.39	0.22–0.67	0.001
Occlusal		1	
**ICDAS baseline**			
ICDAS = 2	2.89	2.31–3.60	<0.001
ICDAS = 3		1	
**Use Fluoride toothpaste**			
Yes	2.39	1.21–4.74	0.012
No		1	
**Baseline dmfs**	0.97	0.96–0.99	0.001

^a^ excluded non-significant variables: gender, mother’s education level, monthly family income, frequency of daily milk feeding, sleep with bottle, frequency of daily snack taking and tooth brushing, and baseline visible plaque index.

**Table 4:** Parental satisfaction on their child’s dental appearance at baseline and the 18-month follow-up.

	Group 1: 38%SDF	Group 2: 5%NaF	*p*-Value
	%(n/N)	%(n/N)	
**Baseline**	N = 147	N = 143	0.504 *
Satisfied	63.9 (94/147)	60.1 (86/143)	
Unsatisfied	36.1 (53/147)	39.9 (57/143)	
**18-month**	N = 122	N = 122	0.896 *
Satisfied	59.8 (73/122)	60.7 (74/122)	
Unsatisfied	40.2 (49/122)	39.3 (48/122)	
***p*-value**	0.775 **	1.00 **	

* Chi-square test; ** McNemar test.

## Data Availability

The datasets generated and/or analyzed during the current study are available from the corresponding author upon reasonable request.
